# Clinical Gait Analysis: Characterizing Normal Gait and Pathological Deviations Due to Neurological Diseases

**DOI:** 10.3390/s23146566

**Published:** 2023-07-20

**Authors:** Lorenzo Hermez, Abdelghani Halimi, Nesma Houmani, Sonia Garcia-Salicetti, Omar Galarraga, Vincent Vigneron

**Affiliations:** 1 SAMOVAR, Télécom SudParis, Institut Polytechnique de Paris, 9 Rue Charles Fourier, 91011 Evry, France; lorenzo.hermez@telecom-sudparis.eu (L.H.); abdelghani.halimi@telecom-sudparis.eu (A.H.); 2 Movement Analysis Laboratory, UGECAM Ile-de-France, 77170 Coubert, France; omar.galarraga@ugecam.assurance-maladie.fr; 3 Informatique, Bio-Informatique et Systèmes Complexes (IBISC) EA 4526, Université Paris-Saclay, 91020 Evry, France; vincent.vigneron@univ-evry.fr

**Keywords:** clinical gait analysis, unsupervised machine learning, normal gait characterization, 3D gait deviation, neurological diseases, Dynamic Time Warping

## Abstract

This study addresses the characterization of normal gait and pathological deviations induced by neurological diseases, considering knee angular kinematics in the sagittal plane. We propose an unsupervised approach based on Dynamic Time Warping (DTW) to identify different normal gait profiles (NGPs) corresponding to real cycles representing the overall behavior of healthy subjects, instead of considering an average reference, as done in the literature. The obtained NGPs are then used to measure the deviations of pathological gait cycles from normal gait with DTW. Hierarchical Clustering is applied to stratify deviations into clusters. Results show that three NGPs are necessary to finely characterize the heterogeneity of normal gait and accurately quantify pathological deviations. In particular, we automatically identify which lower limb is affected for Hemiplegic patients and characterize the severity of motor impairment for Paraplegic patients. Concerning Tetraplegic patients, different profiles appear in terms of impairment severity. These promising results are obtained by considering the raw description of gait signals. Indeed, we have shown that normalizing signals removes the temporal properties of signals, inducing a loss of dynamic information that is crucial for accurately measuring pathological deviations. Our methodology could be exploited to quantify the impact of therapies on gait rehabilitation.

## 1. Introduction

Neurological diseases alter motor functioning in a highly variable way. Upper or lower limbs may be affected simultaneously, as in Paraplegia, or only on one side of the body, as in Hemiplegia. Gait is one of the behavioral modalities in which such motor impairments are visible and can be well characterized, as reported in recent works [1], with different sensors: 3D motion capture [2], IMU and wearable sensors [3,4,5], combined gyroscope and pressure sensor [6] and 3D characterization based on a wearable sensor network [7]. Clinical Gait Analysis tackles the characterization of pathologies through quantitative measures computed on gait signals. These measures quantify the deviation of gait signals from normal gait [4,8,9]. Naturally, the assessment of gait deviations relies on how normal gait is represented as a reference.

In the literature, periodic gait signals of each lower limb are captured during walking and then segmented into gait cycles, corresponding to the signal of one period, defined as the sequence between two events: heel strike and toe-off [10]. Afterwards, gait cycles are normalized into 51 points for further analysis [11,12]. In the framework of angular kinematic data, the reference for normal gait is often based on average normalized curves, for each joint angle, computed on all cycles of the healthy population [7,13]. In such works, the standard deviation or confidence interval of this average curve is computed and reported in graphics. Still, the mean curve remains a unique reference to represent the behavior of the healthy population, and thus to compute the deviation of any cycle from normal gait. Finally, the metric to compute deviations may be a classical Euclidean distance on normalized cycles, or an elastic distance, namely Dynamic Time Warping (DTW) [2,3,4,6,7].

Some works take into account the variance present in the population when computing the reference for a normal gait. This is the case for the Gait Deviation Index [9], which performs Singular Value Decomposition (SVD) on concatenated normalized signals of each joint. SVD is actually performed on the whole population, including individuals with healthy and pathological gaits. The reference for normal gait is then computed in the space of singular vectors (15-dimensional feature space) as the average vector of projected healthy cycles (into 15 features). The deviation of any cycle is then given by the Euclidean distance between its corresponding feature vector and the norm (average vector of projected healthy cycles). We remark that although this approach incorporates gait variability in the generation of the norm, it does not characterize the variance of a healthy gait but that of healthy and pathological gaits altogether (involved in the feature extraction process). On the other hand, as normalized gait cycles are concatenated, signals are processed as static vectors and therefore lose their temporal characteristics.

By contrast, in this work, we propose:

- A refined characterization of normal gait, taking into account: (i) the variance existing among healthy subjects. We identify different Normal Gait Profiles (NGPs), which correspond to real cycles representing the overall behavior of the healthy population (instead of considering an average reference as done in the literature); (ii) the temporal properties of signals, by considering their raw description comparatively to normalized cycles, and using DTW to match different cycles.
- An accurate measure of the deviation from normal gait associated with pathological gait. This measure is based on the identified NGP.
- A new methodology based on Unsupervised Learning, allowing a refined assessment of how different pathologies impact gait. We study the deviations from NGP of three motor impairments, namely Hemiplegia, Paraplegia and Tetraplegia.

More precisely, to extract NGPs, we propose an Unsupervised Learning method that avoids average representatives, namely *K*-medoids [14]. We carry out our study with a very progressive methodology, first on normalized signals with two metrics, Euclidean and DTW and then on raw signals with DTW. We study the number of NGPs required to represent the variance present in our database of 52 healthy subjects. We investigate in each case the impact of the resulting NGP on the deviations shown by pathological signals of 38 patients. The deviations from the NGPs of the three motor impairments (Hemiplegia, Paraplegia and Tetraplegia) are analyzed after being clustered via Hierarchical Clustering into categories. Our approach allows a refined characterization of the gait cycles of each patient into such categories, also stratified by pathology. In this paper, we focus on the analysis of knee joint kinematics in the sagittal plane only (flexion/extension) because of its major role in maintaining stance stability on this plane, being characteristic of normal gait [10].

Section 2 describes our database and its acquisition protocol, as well as the methodology proposed in this work. In Section 3, we present our results, and in Section 4, we discuss these alongside the prospects for future work.

## 2. Materials and Methods

### 2.1. Database Description

For this retrospective study, we exploited angular kinematic data acquired during a spontaneous gait task from 52 healthy subjects and 38 patients suffering from neurological diseases. Data were collected at the Movement Analysis Laboratory of Coubert Rehabilitation Center, at UGECAM Ile-de-France. Each participant was informed that his/her data could be used for research purposes, and no participants were opposed to the utilization of his/her data.

Data acquisition was performed with the optoelectronic Codamotion system, integrating four CX1 cameras placed in each corner of the laboratory. The system allows for recovering angles’ kinematics during gait for five joints (pelvis, hip, foot, ankle and knee) in the three planes (sagittal, frontal and transverse), with a sampling rate of 100 Hz.

Persons were asked to walk naturally for 10 m in a straight line and on flat ground, with a spontaneous speed. This process was repeated 5 times on average, with each corresponding to one trial.

The recruited healthy subjects were young adults (students or laboratory staff) ranging from 18 to 41 years old with an average age of 22.62 years old. Among them, 34 were female (65.38%) and 18 were male (34.62%). They had no disease affecting motor function. Table 1 reports more details about the recruited population.

The 38 pathological patients were adults ranging from 21 to 75 years old with an average age of 42.82 years old. Among them, 25 were male (65.79%) and 13 were female (34.21%). These patients were followed-up at the Coubert Rehabilitation Center for motor problems caused by neurological diseases, such as Cerebral Palsy, Traumatic Brain Injury, Spinal Cord Injury, Stroke, or Multiple Sclerosis. These diseases are often the cause of paralysis or motor impairments affecting one or more limbs of the upper and/or lower body, e.g., Hemiplegia (HP), Tetraplegia (TP) or Paraplegia (PP). Table 2 summarizes the number of patients with each disease causing different motor impairments, namely HP, PP and TP.

During acquisitions, 12 patients walked with a technical aid: 10 patients walked with a cane and 2 patients with a tripod cane. Two other patients walked with platform shoes, one with a rollator and one with two walking sticks. Other information is also available, such as which side is affected by the disease and on which side the cane is held.

### 2.2. Data Pre-Processing

**Figure 1:**
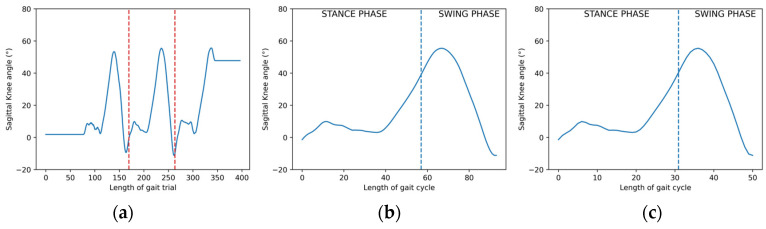
Knee angular kinematics (flexion/extension) in the sagittal plane: (**a**) a periodic sequence of one trial; (**b**) a raw segmented knee cycle; (**c**) the normalized segmented knee cycle. Segmentation was performed with the high-pass algorithm [15] and controlled by an expert. Red dotted lines define the beginning and end of a gait cycle. Blue dotted lines split a gait cycle into its two phases, Stance and Swing phases.

The angular kinematics of each joint, captured during each gait trial, is a periodic signal consisting of different consecutive cycles, defined between the initial contact event and the terminal swing event (see Figure 1a). This complete captured signal was segmented into gait cycles, automatically detected with the high-pass algorithm [15] and controlled by an expert (see Figure 1b).

As mentioned in the Introduction, we focused on the analysis of the knee joint kinematics in the sagittal plane only (flexion/extension). Gait cycles were either used raw (see Figure 1b) or normalized into 51 points (i.e., 1 point for every 2% of the gait cycle), as shown in Figure 1c.

The number of cycles was not the same for all trials and differed for each patient. The total number of knee sagittal cycles used in our experiments was 872 cycles, among which 526 cycles belonged to healthy subjects and 346 cycles belonged to patients (162 for HP, 106 for TP, and 78 for PP patients).

### 2.3. Methods

For a refined characterization of normal gait, we adopted an unsupervised approach based on an elastic metric to match different cycles. We performed a *K*-medoids algorithm on all healthy cycles in order to retrieve reference normal gait cycles, denoted in the following sections as “Normal Gait Profiles”. Then, we studied the deviation of pathological gait cycles from the obtained Normal Gait Profiles (NGPs), according to the three types of motor impairments available in the dataset.

#### 2.3.1. Identification of Normal Gait Profiles

The *K*-medoids algorithm is an unsupervised method that divides a population of *N* samples (cycles) into *K* clusters, according to a given metric [14]. This algorithm is based on the principle of minimizing the sum of the distances between each sample in the cluster and its representative sample (called a medoid). In our case, each cluster was represented by a medoid corresponding to an existing cycle (in our dataset) that was the closest on average to all the other cycles belonging to such a cluster. We chose this unsupervised method to obtain a representative cycle per cluster that corresponds to a real gait cycle of one subject.

The final partition of samples was very sensitive to the initialization of the *K*-medoids. The classical version of the algorithm randomly selects *K* initial medoids from the dataset; if the first medoids are well chosen, the algorithm is more likely to converge to a better solution, resulting in relevant clusters according to the data distribution. For this reason, we used in this work the heuristic *K*-medoids++ initialization strategy. It selects the first medoid randomly, and subsequent medoids are the most distant from the previously selected ones. This approach allowed us to obtain a better representation of the data due to the extracted medoids, which adequately covered the whole space.

Additionally, the classical version of *K*-medoids usually exploits the Euclidean distance as a dissimilarity metric. However, in our case, this distance requires all cycles to have the same length, and does not take into account time shifts and intrinsic variations when comparing two cycles.

**Figure 2:**
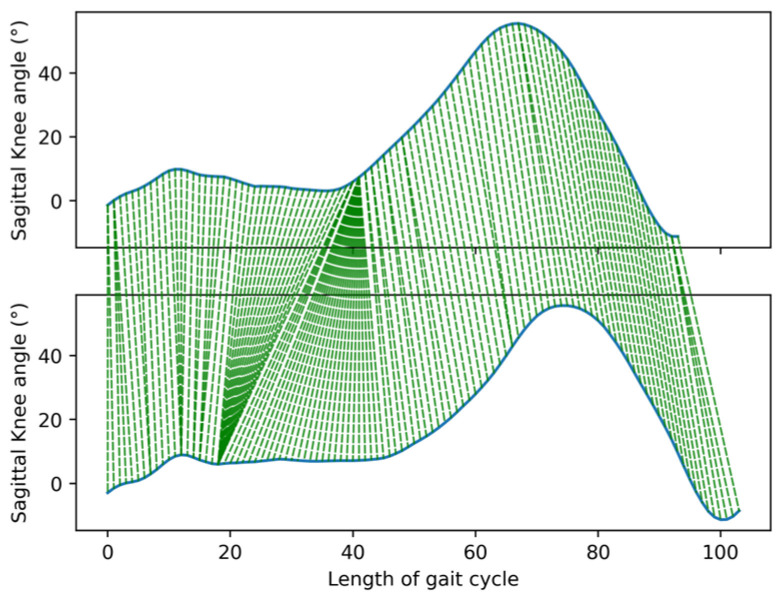
DTW matching between two healthy knee angular signals of different lengths without time constraints.

In our work, we faced a strong variability between individuals and even within the cycles of the same individual. To overcome the above-mentioned limitations of Euclidean distance, we integrated in the *K*-medoids algorithm an elastic distance, namely Dynamic Time Warping (DTW,) as a measure of the dissimilarity between two cycles [16]. DTW relies on finding the best warping path to assign two time signals, by minimizing the cumulative distance between the assigned points in the two signals. As illustrated in Figure 2, DTW accounts for time distortions between two signals (cycles) of different lengths.

We performed *K*-medoids on the 526 healthy gait cycles in order to automatically extract *K* Normal Gait Profiles, denoted as $m_{1}$, *…*, $m_{K}$. Then, we used these NGPs as references to quantify the deviations of pathological gait cycles from normal gait.

#### 2.3.2. Measuring the Deviation of Pathological Gait Cycles from Normal Gait

We computed DTW distances of any cycle (healthy or pathological) in the dataset to obtain the *K* Normal Gait Profiles ($m_{1}$, …$,m_{j}$, ${..,m}_{K}$). Each cycle $c_{i}$ is then represented by a *K*-dimensional vector $D_{i}$, where:

(1) $$D_{i}\;=\;[D_{i,1},\ldots ,D_{i,K}],$$

with $D_{i,j}$ = *DTW* ($c_{i}$,$m_{j}$) *≥* 0. This vector is used to quantify the deviation between a given gait cycle and Normal Gait Profiles.

To analyze the deviation of pathological cycles, according to the three types of motor impairments, we applied Agglomerative Hierarchical Clustering (AHC) [17] on the *K*-dimensional vectors $D_{i}$ associated with each cycle.

**Figure 3:**
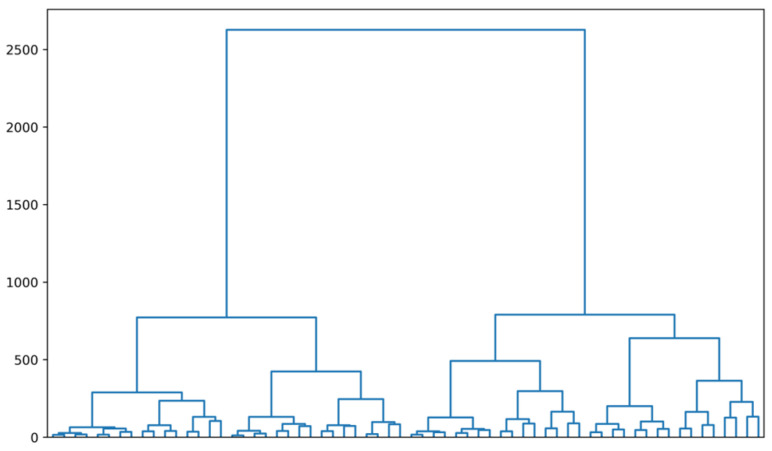
Dendrogram obtained with Agglomerative Hierarchical Clustering using Ward’s linkage.

We chose the AHC algorithm because it does not require fixing a priori the number of clusters and it is initialized by considering each data cycle as a singleton cluster. Moreover, this algorithm allows for analyzing the progressive data aggregation into clusters, thanks to the dendrogram, as displayed in Figure 3.

There are several possible linkage criteria (how the distance between two clusters is defined) that can be used to perform Hierarchical Clustering. We chose Ward’s linkage because it tends to create tight, well-separated clusters that are robust to outliers [17].

## 3. Results

For the experiments, we first considered the cycles normalized into 51 points, as usually done in the literature [11,12], and performed *K*-medoids on the healthy population with Euclidean distance on the one hand, and with DTW distance on the other hand. Then, for both metrics, we studied the deviation of healthy and pathological cycles from the retrieved Normal Gait Profiles (NGP). After that, we followed the same methodology and compared the results when using raw gait signals (without normalization). Finally, we investigated the influence of the number of NGPs on the representation of the healthy population and deviation assessment.

### 3.1. Study on Normalized Gait Cycles

#### 3.1.1. Retrieving Normal Gait Profiles

**Figure 4:**
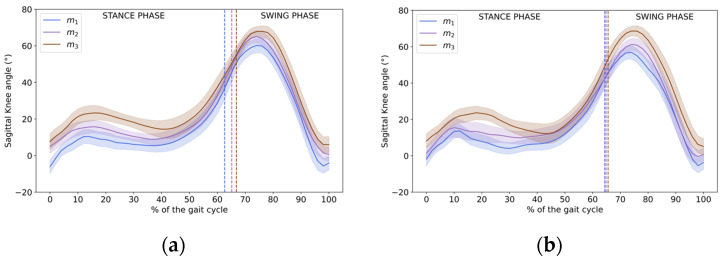
The three NGP (cycles) representing healthy subjects in our dataset, extracted with the *K*-medoids algorithm using: (**a**) Euclidean distance and (**b**) DTW distance. For each NGP, reported in one color, Stance and Swing phases are split thanks to a dotted line in the corresponding color.

We applied *K*-medoids, as explained in Section 2.3.1, by fixing a priori *K* = 3 for a first insight; this allowed us to consider two extreme behaviors and one intermediate one. Figure 4 shows the three NGPs (medoids) on 51 points, as obtained with Euclidean distance (Figure 4a) and DTW (Figure 4b). The signals are displayed in terms of percentage of gait cycle.

We first observed that the three medoids obtained with the Euclidean distance (see Figure 4a) showed a similar smooth shape, but differed in their amplitudes, especially during the loading response in the stance phase [10]. Also, we noted a lag in the transitions between the stance and swing phases for the three NGPs, ranging from 61.4% of the cycle for $m_{1}$ to 65.5% of the cycle for $m_{3}$, as indicated in Table 3 (foot-off event). Additionally, the three cycles showed a progressive increase in the knee angle amplitude from $m_{1}$ to $m_{3}$. With the DTW metric (see Figure 4b), the retrieved medoids showed more diversity during loading response and differed from those obtained with the Euclidean distance. Table 3 and Table 4 show the metadata associated with each NGP for both metrics. We did not observe a significant difference between the medoids in the two tables, but noted a difference between the three medoids in DTW in terms of speed and foot-off event.

With DTW, the three NGPs seemed to capture the diversity that naturally exists in the healthy population: we noted, particularly in the stance phase, that the retrieved NGPs were irregular and showed time shifts between one another. Additionally, we noted, as can be seen in Figure 4b, that the transitions between the stance and swing phases of the three NGPs appeared between 63.1% and 64.3% of the cycle, as indicated by the foot-off event in Table 4. This reflects the potential of DTW for time-aligning two signals at important transitions comparatively to Euclidean distance.

#### 3.1.2. Studying the Deviations from Normal Gait Profiles

**Figure 5:**
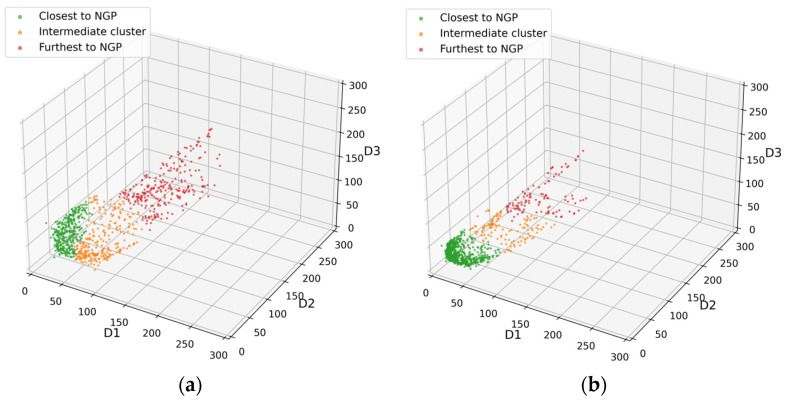
Three-dimensional representations of clusters in three colors (green, orange and red) with (**a**) Euclidean distance and (**b**) DTW distance.

**Figure 6:**
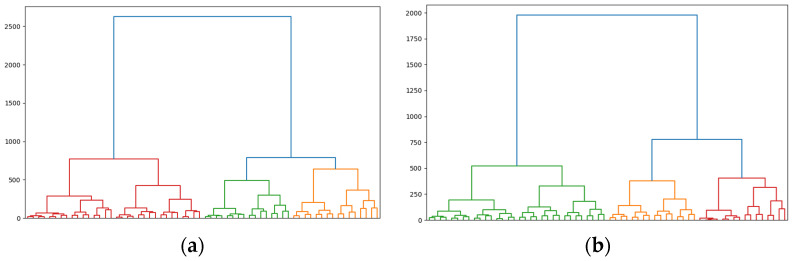
Dendrograms obtained using AHC on all the cycles, which are represented by their associated 3D distance vectors to the three NGPs, with (**a**) Euclidean distance and (**b**) DTW distance. Colors represent the three clusters obtained by AHC.

As described in Section 2.3.2, we performed AHC on the three-dimensional vectors representing the deviations of each cycle from the three NGPs. We visualize in Figure 5 the resulting three clusters with AHC in the three-dimensional space. Green gait cycles are the closest to the three NGPs, the red cycles are the most distant, and the orange ones show an intermediate behavior. We show in Figure 6 the obtained dendrograms and incorporate the color code of Figure 5.

In the case of DTW distance, we observed a linear trend in the distribution of clusters in the 3D space: the orange cluster falls exactly between the green and red clusters. This was clearly not the case with the Euclidean distance. Additionally, the green cluster shows less variance within the DTW metric (see Figure 5b compared to Figure 5a).

Consequently, we can see in the dendrograms that with the DTW metric (see Figure 6b), the green cluster (the closest to the NGP) is isolated in a unique branch, while the orange and red clusters are agglomerated together in the other branch. With Euclidean distance (see Figure 6a), the green cluster (the closest to the NGP) is agglomerated together with the orange one (intermediate distance to the NGP). In this case, the red cluster representing the most distant cycles to normal gait is isolated; this is the opposite trend to that observed with DTW metric.

**Figure 7:**
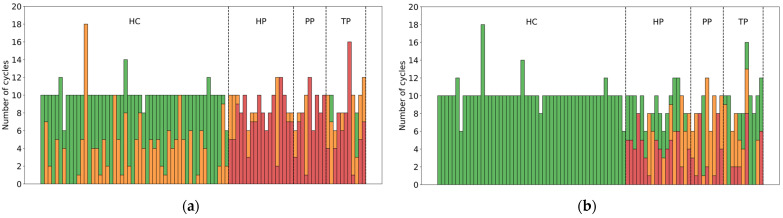
Distribution of cycles for each person (a bar represents one person) within the three clusters with (**a**) Euclidean distance and (**b**) DTW distance. Cycles in green are the closest to NGPs, followed by cycles in orange and then in red (the most distant to NGPs). Persons are grouped according to their class: HCs for healthy controls, HP for Hemiplegic, PP for Paraplegic and TP for Tetraplegic patients.

We enrich our analysis by displaying, in Figure 7, the distribution of cycles per person in clusters. Each person was described by a vertical bar, accounting for the number of cycles of this person in each cluster, maintaining the same color code (green, orange and red). For better readability of the graph, healthy controls (HCs) are grouped on the left and patients are grouped by motor impairment on the right, sorted as follows: Hemiplegia (HP), Paraplegia (PP) and Tetraplegia (TP).

With DTW distance (see Figure 7b), all healthy cycles are grouped into the closest cluster to the NGP (green cluster). On the other hand, with Euclidean distance (see Figure 7a), the gait cycles of the HCs are assigned to two clusters (green and orange). Additionally, with Euclidean distance, we noticed that most pathological cycles, independently of motor impairments, are grouped in the red cluster (the most distant to the NGP). On the contrary, with DTW, we observed different distributions in clusters between motor impairments (HP, PP, TP), and in each impairment, an increased stratification of cycles into the three clusters. When we focus on HP patients with DTW, most cycles are assigned to two clusters: 13 HP patients among the 18 have some of their cycles in the green cluster and the remaining ones are mostly in the red cluster. This is in accordance with the lateral impact of Hemiplegia: the healthy side is close to the NGP (green cluster), whereas the cycles of the impacted side seemed to be considered strongly (red) or slightly (orange) impacted. For TP patients, we noted more green cycles and fewer red cycles; this is in accordance with the fact that these TP patients had incomplete Tetraplegia.

**Figure 8:**
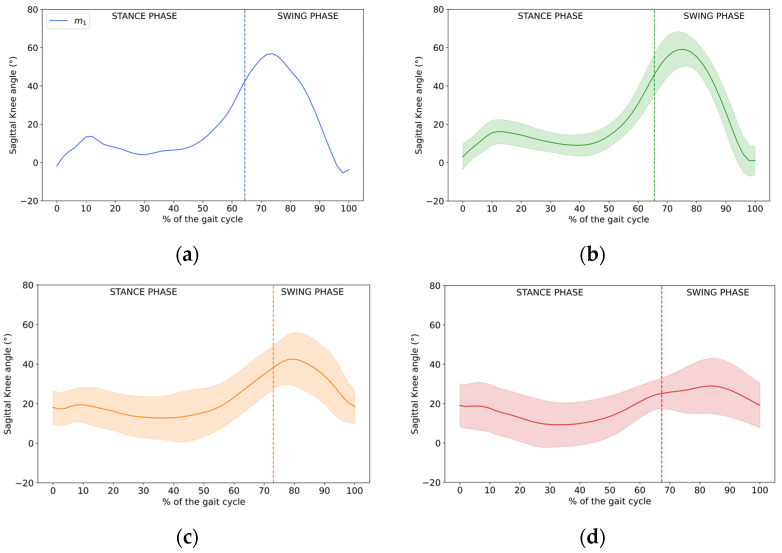
Examples of: (**a**) one NGP ($m_{1})$, (**b**) average curve of all cycles belonging to the green cluster, (**c**) average curve of all cycles belonging to the orange cluster and (**d**) average curve of all cycles belonging to the red cluster. The standard deviation is also reported along with the average. For the NGP in (**a**) and the average curve of each cluster in (**b**–**d**), each reported in one color, Stance and Swing phases are split thanks to a dotted line in the corresponding color.

To illustrate the progressive degradation of gait cycles from NGPs, we display in Figure 8 one NGP (see Figure 8a) and the average curve of all the cycles belonging to each cluster, in its respective color (green, orange and red), along with the standard deviation.

The progressive degradation of gait cycles from the green cluster to the orange one and finally the red one, as well as the progressive increase in the standard deviation, can be seen between Figure 8b–d. Given these trends, we may consider the cycles in the orange and red clusters as being, respectively, slightly and severely impacted by the disease.

All these findings point out that DTW metric is more effective than Euclidean distance in identifying NGPs and characterizing the deviations from normal gait. Since DTW can measure the dissimilarity between sequences of different lengths, we followed the same methodology considering raw sequences versus normalized ones, both to identify NGPs and characterize the deviations from normal gait.

### 3.2. Impact of Normalization on Gait Characterization

**Figure 9:**
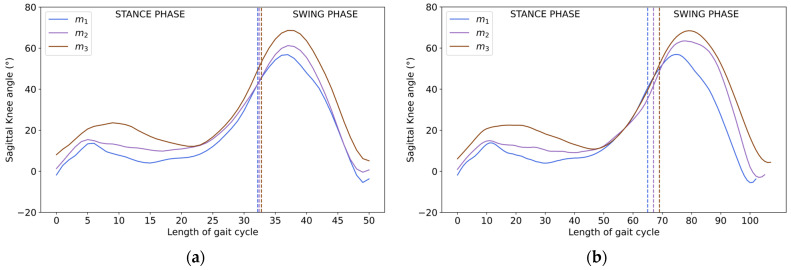
The three NGPs (cycles) representing healthy subjects in our dataset, extracted with the *K*-medoids algorithm using DTW on (**a**) normalized signals and (**b**) raw signals. For each NGP, reported in one color, Stance and Swing phases are split thanks to a dotted line in the corresponding color.

We investigated the effect of normalization by considering on the one hand raw signals and on the other hand normalized sequences (normalized into 51 points). Figure 9 shows the three NGPs obtained with DTW in each case. We display the cycles in terms of length instead of percentage of the gait cycle to better assess normalization effects. Of note, in the rest of the paper, the cycles are all displayed in terms of length (number of points) for a better visualization of raw signals.

The respective metadata of the obtained NGPs are reported in Table 4 and Table 5. We noticed that the three NGPs in both cases displayed the same metadata trend, even if they were not the same cycles (except for $m_{1}$).

Figure 9 shows that the transitions from stance to swing phases were more variable between the three NGPs in the case of raw signals (see Figure 9b) compared to normalized signals (see Figure 9a). This highlights, on the one hand, that DTW tolerates temporal variabilities during alignment between two signals of different lengths; on the other hand, this reflects that normalizing signals into 51 points (as in Figure 9a) induces slight alterations in the signal, which tend to reduce the temporal lag between pairs of signals.

**Figure 10:**
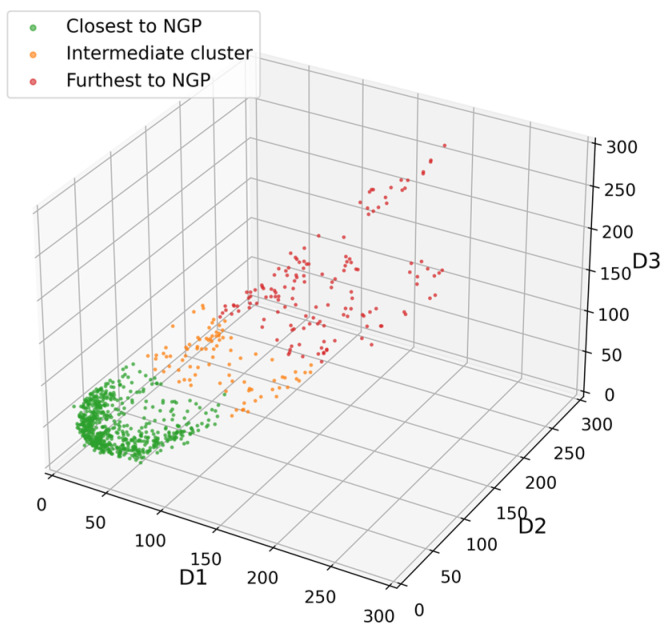
Three-dimensional representations of clusters in three colors (green, orange and red) for raw cycles.

**Figure 11:**
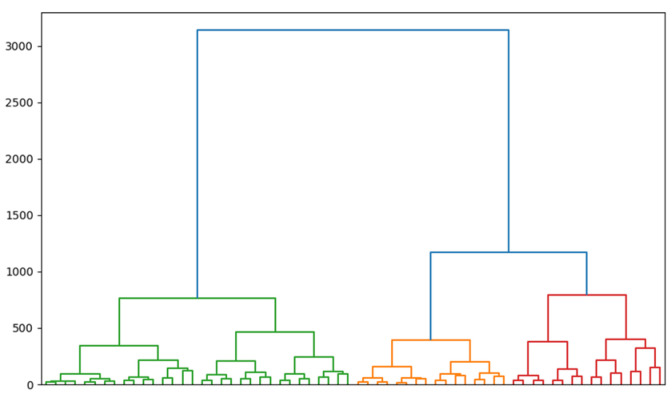
Dendrogram obtained using AHC for all the cycles, which are represented by their associated 3D distance vectors to the three NGPs, for raw cycles. Colors represent the three clusters obtained by AHC.

Figure 10 displays the deviations of healthy and pathological cycles from the NGPs shown in Figure 9b. We noticed that the linear trend observed in Figure 5b (for normalized cycles) is maintained in Figure 10 (for raw signals) but with a higher dispersion of cycles in the 3D space. The dispersion is especially increased for the orange and red clusters. By comparing the dendrogram in Figure 11 to that in Figure 6b, we observe the same trend for both types of signals, but with higher aggregation distances for raw signals.

**Figure 12:**
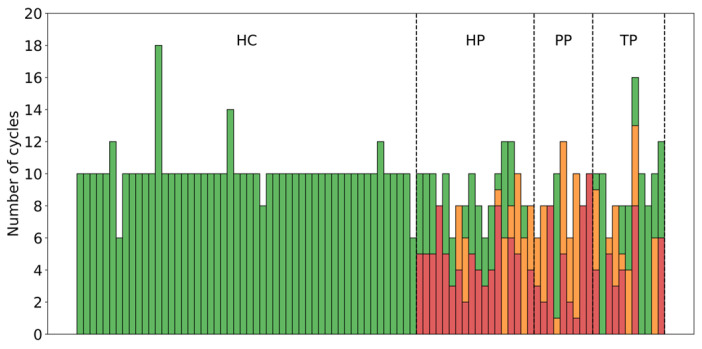
Distribution of cycles for each person (a bar represents one person) within the three clusters for raw cycles. Cycles in green are the closest to the NGP, followed by cycles in orange and then in red (the most distant to the NGP). Persons are grouped according to their class: HCs for healthy controls, HP for Hemiplegic, PP for Paraplegic and TP for Tetraplegic patients.

Figure 12 presents the cycles that are assigned to the three clusters relative to their labels. Compared to Figure 7b (for normalized cycles), the difference concerns only patients, since all healthy cycles remained assigned to the green cluster, as expected. For HP patients, in most cases, the cycles assigned to the red cluster corresponded to the impacted side. For such patients, we clearly observe in Figure 12 that some of their cycles were assigned to the green cluster, accounting for the non-impacted side, while the other cycles were assigned to the most distant cluster to the NGP (red).

**Figure 13:**
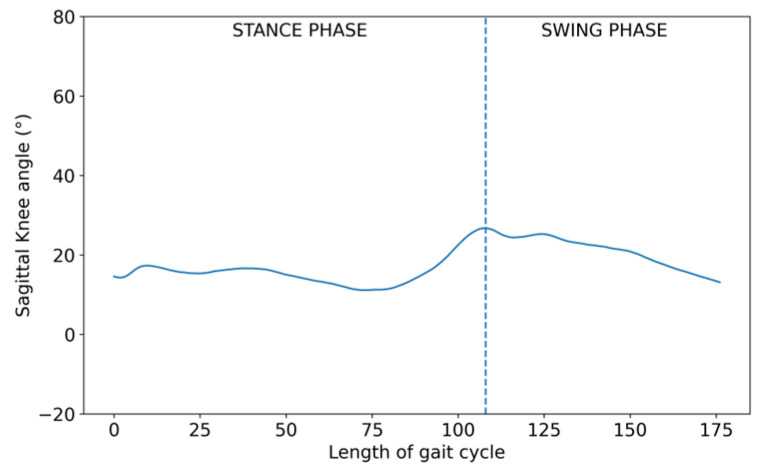
Gait cycle assigned to the green cluster after being normalized and to the red cluster when kept raw.

Moreover, we noticed that PP patients had more cycles in the red cluster for raw signals, accounting for a more severe impact of the disease on gait functioning. Finally, for TP patients, their characterization was roughly the same for both types of signals, except for three patients who had more cycles assigned to the red cluster for raw signals. More precisely, for pathological cycles, we observed that one green cycle became red when considering the raw signal. To aid understanding, we show this cycle in Figure 13. Visually, it is clear that this cycle was completely different from the NGP (displayed in Figure 9).

**Figure 14:**
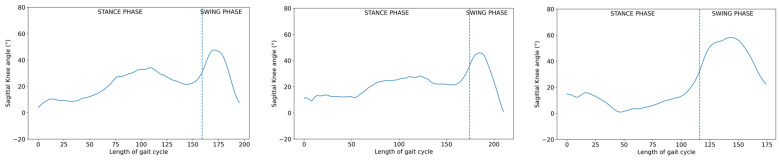
Three gait cycles assigned to the green cluster after being normalized and to the orange cluster when kept raw.

Also, three green cycles became orange when considering raw signals. Figure 14 displays these cycles; we can confirm that the associated signals are visually different from the NGPs, especially in the stance phase.

**Figure 15:**
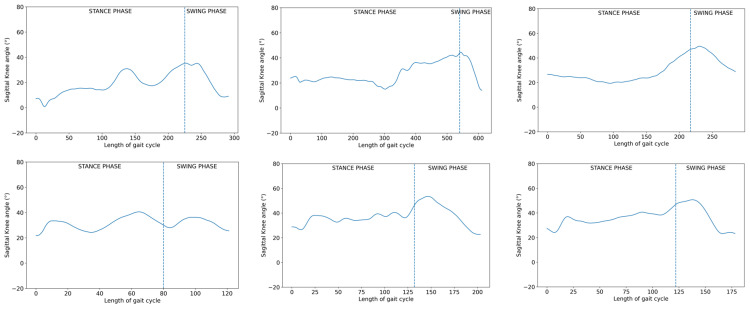

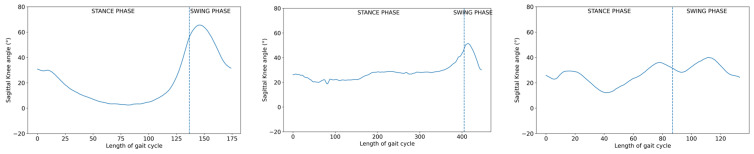
Examples of raw gait cycles assigned to the red cluster, whereas previously they were assigned to the intermediate orange cluster (when normalized). For each cycle, Stance and Swing phases are split thanks to a dotted line.

Finally, 36 orange cycles became red when considering their raw description. Some examples are displayed in Figure 15. Again, we noticed the significant quality degradation in such gait cycles.

For all these reasons, we infer that exploiting raw signals allows for capturing more local variations in the signal, as DTW time-aligns each cycle to the NGP, whereas the normalization process tends to smooth such local variations. We thus conclude that keeping the cycles as raw signals allows a more accurate characterization of pathological cycles in terms of their deviation from normal gait.

### 3.3. Influence of the Number of Normal Gait Profiles on Gait Characterization

In the previous section, we reported the results considering three Normal Gait Profiles. In this section, we study the influence of the number of Normal Gait Profiles (NGP), *K*, on measured deviations from normal gait, using only raw signals.

**Figure 16:**
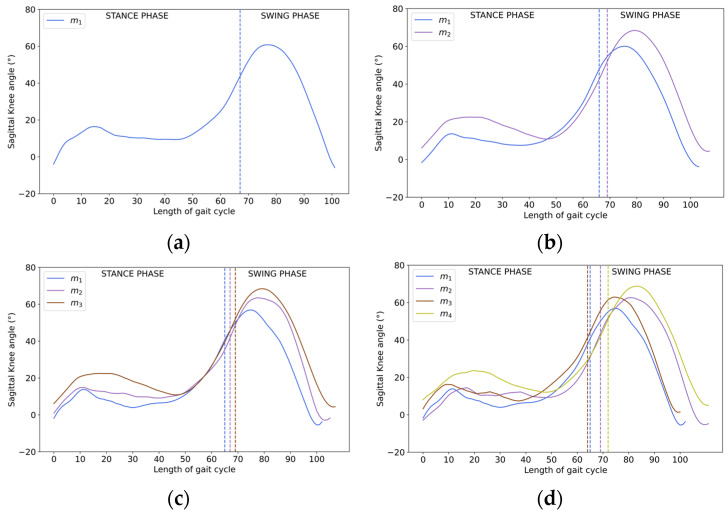
The retrieved NGPs considering (**a**) one reference (*K* = 1), (**b**) two references (*K* = 2), (**c**) three references (*K* = 3) and (**d**) four references (*K* = 4) to represent the variability of normal gait. For each NGP, reported in one color, Stance and Swing phases are split thanks to a dotted line in the corresponding color.

Figure 16 shows the obtained NGP for a *K* varying from 1 to 4, in terms of the length of the gait cycle since we are considering the raw description of signals. We can observe that when *K* is varied, the healthy cycles representing normal gait are different in each case, and when *K* is progressively increased, they take into account the variability present in the healthy population.

**Figure 17:**
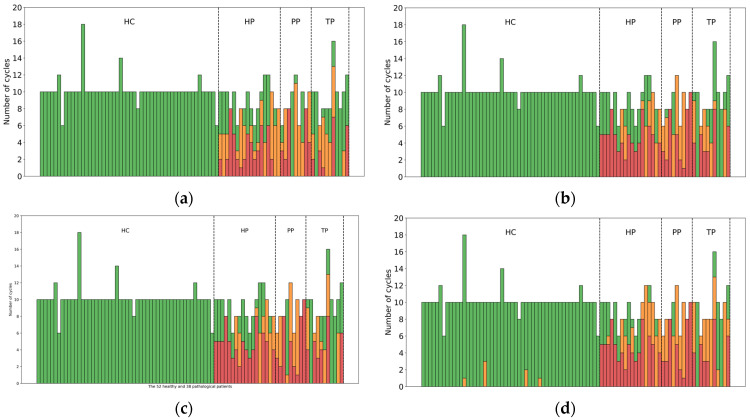
Distribution of raw cycles within the three AHC clusters considering (**a**) one reference (*K* = 1), (**b**) two references (*K* = 2), (**c**) three references (*K* = 3) and (**d**) four references (*K* = 4).

Figure 17 displays the distribution of cycles in the three AHC clusters, based on their deviation from the NGP. We can observe that until *K =* 3 NGPs, the characterization of healthy cycles was the same. For *K* = 4, however, this characterization began to degrade as some healthy cycles were assigned to the orange intermediate cluster. This is because we increased the diversity of the NGPs with *K* = 4.

**Figure 18:**
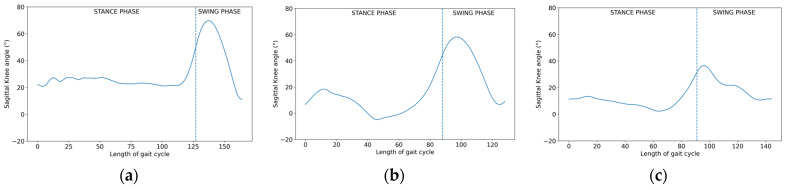
Examples of (**a**) a cycle assigned to the green cluster with *K* = 2 and to the orange one with *K* = 3, (**b**) a cycle assigned to the orange cluster with *K* = 2 and to the green one with *K* = 3, and (**c**) a cycle assigned to the orange cluster with *K* = 2 and to the red one with *K* = 3. For each cycle, Stance and Swing phases are split thanks to a dotted line.

When *K* = 1, we can see in Figure 17a that pathological cycles tended to be assigned to the intermediate orange cluster. In fact, due to the variance existing in the healthy population (variability between individuals and within an individual), one reference is clearly not sufficient to characterize the population. As a consequence, pathological cycles become nearer to the NGP representing normal gait. Additionally, we noticed that when *K* = 1, the distinction between the impacted and the non-impacted sides in HP patients was lost. When comparing results with *K* = 2 and *K* = 3, we noticed a slight difference between them. Indeed, cycles assigned to the red cluster were the same, except one (shown below in Figure 18c). Finally, the number of cycles belonging to the same cluster was 856 out of 872 (i.e., 98.2% of the total cycles). The remaining cycles were assigned to the orange or to the green clusters depending on *K*. To aid understanding, we show in Figure 18 examples of these cycles.

We can conclude that clustering with *K* = 3 seems more effective than clustering with *K* = 2. More precisely, we see in Figure 18a that the trend of this signal is not typical of normal gait during the stance phase. Instead, in Figure 18b, the signal follows a closer trend to that of normal gait. Finally, it is clear that the signal shown in Figure 18c does not follow the trend of normal gait and is quite degraded. These three examples demonstrate that the assignments performed with three NGPs are more accurate.

### 3.4. Considering an Average Cycle as a Reference for Normal Gait

**Figure 19:**
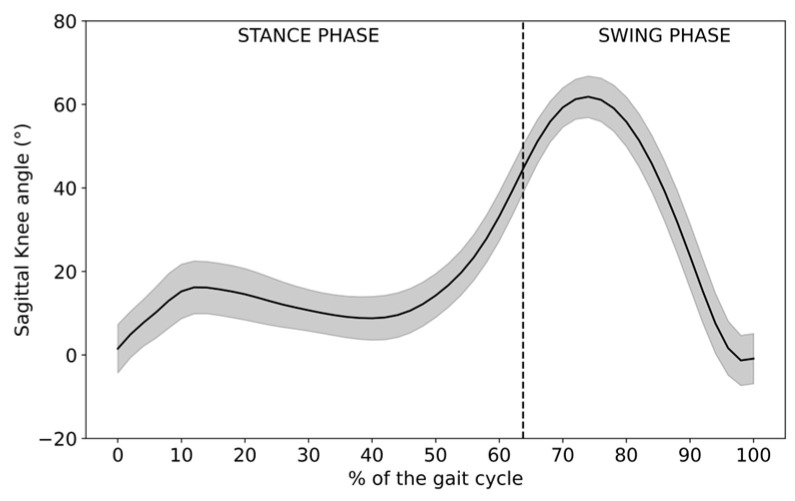
The retrieved average cycle for normal gait for normalized cycles. Stance and Swing phases are split thanks to a dotted line.

To enhance our analysis, we considered as a reference for normal gait the average cycle for the whole healthy population, as is usually done in the literature. To this end, we normalized cycles into 51 points and computed the reference displayed in Figure 19 in terms of percentage of gait cycle.

**Figure 20:**
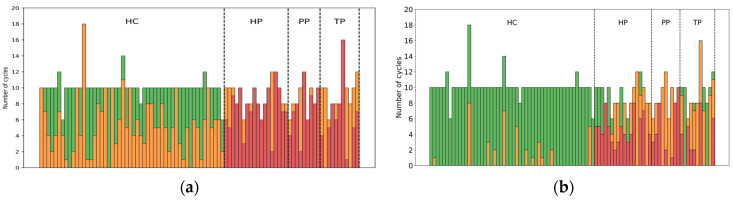
Distribution of normalized cycles within the three AHC clusters considering (**a**) average cycle as a reference and Euclidean distance to compute the deviations, (**b**) average cycle as a reference and DTW to compute the deviations, and considering raw cycles.

Then, we computed for each healthy and pathological cycle its deviation from this average reference with the Euclidean distance on the one hand and DTW metric on the other hand. After that, we performed, in both cases, AHC on the resulting distances. Figure 20a,b display the distribution of cycles per person to the three obtained clusters with the Euclidean distance and DTW, respectively.

When considering the average as a reference, we noticed a degradation in the characterization of healthy subjects and that of patients as well, as shown in Figure 20a,b, comparatively to when we considered three NGPs as references (see Figure 17c). In fact, using the average of normalized cycles (into 51 points) as the reference for normal gait leads to categorizing healthy subjects into two clusters (green and orange). This is because an average reference cannot capture the diversity of the healthy population well.

Additionally, with the Euclidean distance (see Figure 20a), most pathological cycles were assigned to the extreme category in terms of deviation (red cluster). With DTW (see Figure 20b), we observed a better characterization of healthy cycles (the majority belonged to the green cluster) and pathological ones (stratified into three clusters). However, this improvement in characterizing individuals with DTW remains less effective compared to when we consider three NGPs and raw signals. Indeed, with our methodology (see Figure 17c), we observed that: (i) all healthy cycles were in the green cluster, meaning that three NGPs managed to capture the variability existing in the healthy population; (ii) for Hemiplegic patients, there was a better distinction between impacted and non-impacted sides; and (iii) for Tetraplegic patients, more cycles were assigned to the green cluster, in accordance with the fact that the TP patients in our dataset had incomplete Tetraplegia.

These results confirm that considering an average waveform as a reference for normal gait leads to an information loss in representing the healthy population. Thereby, it affects the quality of deviation measures for pathological cycles, and consequently, the characterization of gait pathologies.

## 4. Discussion and Conclusions

Comparatively to the literature, which usually refers to one average Normal Gait Profile [7,13] (per joint), the results of our research indicate that three NGPs are necessary to comprehensively characterize normal gait, based on knee angular kinematics in the sagittal plane. It is noteworthy that we obtained three NGPs, although the healthy subjects under study were young and of a similar age. Therefore, we expect the number of NGPs to be increased when considering databases of healthy subjects spanning larger age ranges.

Also, for an accurate characterization of pathological patients, we have proposed that DTW distance be used to compute the dissimilarity between two cycles. We have shown that DTW outperforms the Euclidean distance in characterizing patients’ normalized signals into 51 points. In addition, as DTW allows for measuring the dissimilarity between sequences of different lengths, we computed the deviations of raw signals from NGP. The results have shown that in this case, the method captures the intrinsic local variations in the signal induced by the disease well.

The results allowed us to first distinguish healthy cycles from pathological ones and also to categorize pathological gait cycles into three clusters. In addition, we have shown the capability of our proposed method to distinguish, for Hemiplegic patients, the impacted side from the non-impacted one, and to characterize the severity of motor impairment for Paraplegic patients. Concerning Tetraplegic patients, different profiles appear in terms of impairment severity. As our dataset contains incomplete-TP patients, we found that some of them show a motor behavior close to normal gait.

Our work is novel in two aspects. First, the majority of works in the literature characterize normal gait by considering one profile, which is the average waveform computed over the normalized consecutive cycles of all healthy individuals [7,13]. By contrast, we identified different NGPs corresponding to real cycles in our dataset to represent the diversity of behaviors in the healthy population. To this end, we have shown that it is crucial to keep the temporal properties of raw signals and to use an unsupervised approach using the DTW metric.

The second aspect is how the deviation from NGPs is measured. The Gait Deviation Index (GDI) is a widely used score in the literature that quantifies the deviation from a norm [9]. This score uses normalized cycles of different joints concatenated in a high-dimensional vector. In this work, we demonstrate the importance of keeping the temporal characteristics of gait signals when measuring the deviation of pathological cycles from NGPs. Our methodology considers raw signals and deviations from NGPs measured with DTW. Then, the obtained deviations are clustered with Hierarchical Clustering in a 3D space, since we consider three NGPs. Our findings show that this methodology leads to a refined characterization of pathologies, highlighting different trends in each, especially in Hemiplegic patients, for whom we distinguish between the impacted and non-impacted sides.

Our methodology has the advantage of being totally explainable and interpretable, even through visualization of deviations in a 3D space. This aspect is of high interest for medical staff since they can better understand the outcomes of the method by observing the signals and their temporal properties. Finally, our method allows for addressing several pathologies simultaneously.

Our proposal suffers from some limitations. One of the main limitations concerns the database used, which lacks diversity in terms of age representation for healthy subjects. In future work, we will study a more comprehensive healthy population to more accurately characterize normal gait across age and better assess deviations related to pathologies. Moreover, we could evaluate the potential of our methodology for quantifying the impact of therapies in the context of gait rehabilitation, independently of the disease, by measuring the deviation from NGPs before and after therapy on pre-treatment and post-treatment waveforms. In addition, our research has focused primarily on the knee joint. It is necessary to consider other joints, such as the hip and ankle, in order to attain a more complete understanding of the biomechanics involved in normal and pathological gait.

## Figures and Tables

**Table 1:** Descriptive statistics for healthy subjects and patients.

	Healthy Subjects	Pathological Patients
Number of patients	52	38
Female	34	13
Age (Mean ± Std)	22.62 ± 3.89 (years old)	46.82 ± 12.93 (years old)
Height (Mean ± Std)	1.71 ± 0.09 (m)	1.70 ± 0.10 (m)
Weight (Mean ± Std)	65.28 ± 10.77 (kg)	71.06 ± 13.99 (kg)
Speed	1.20 ± 0.14 (m/s)	0.52 ± 0.24 (m/s)
Stride time	1.08 ± 0.06 (s)	1.81 ± 0.84 (s)
Foot-off event	62.51 ± 1.9 (% of gait cycle)	70.08 ± 8.0 (% of gait cycle)
Stance phase amplitude min	1.3 ± 5.6 (°)	7.0 ± 9.5 (°)
Stance phase amplitude max	38.4 ± 6.3 (°)	36.7 ± 11.4 (°)
Swing phase amplitude min	−2.1 ± 6.1 (°)	15.2 ± 9.9 (°)
Swing phase amplitude max	62.2 ± 4.9 (°)	47.5 ± 14.7 (°)

**Table 2:** Number of patients with Hemiplegia, Tetraplegia and Paraplegia caused by each disease.

Neurological Disease	Hemiplegia	Tetraplegia	Paraplegia	Total
Cerebral Palsy	0	0	3	3
Multiple Sclerosis	1	3	5	9
Spinal Cord Injury	5	8	1	12
Stroke	8	0	0	8
Traumatic Brain Injury	4	0	0	4
Total	18	11	9	38

**Table 3:** Metadata of the three NGPs obtained with Euclidean distance for normalized gait cycles.

NGP	Gender	Age	Height	Weight	Side	Speed (m/s)	Foot-Off Event (% of Cycle)	Amplitude Range Stance Phase (in °)	Amplitude Range Swing Phase (in °)
$m_{1}$	F	20	1.83	62.0	R	1.16	61.4	[−6.1, 28.8]	[−5.7, 60.2]
$m_{2}$	F	21	1.69	51.0	L	1.09	63.9	[4.9, 45.3]	[0.6, 65.1]
$m_{3}$	F	20	1.68	58.9	L	1.06	65.5	[7.6, 47.4]	[6.0, 68.0]

**Table 4:** Metadata of the three NGPs obtained with DTW distance for normalized gait cycles.

NGP	Gender	Age	Height	Weight	Side	Speed (m/s)	Foot-Off Event (% of Cycle)	Amplitude Range Stance Phase (in °)	Amplitude Range Swing Phase (in °)
$m_{1}$	F	20	1.61	65.0	L	1.23	63.1	[−1.8, 35.5]	[−5.4, 56.9]
$m_{2}$	F	27	1.66	64.7	R	1.00	63.6	[1.3, 36.7]	[−0.4, 61.2]
$m_{3}$	M	22	1.83	68.1	L	1.17	64.3	[8.1, 48.1]	[5.1, 68.6]

**Table 5:** Metadata of the three NGPs obtained with DTW distance for raw gait cycles.

NGP	Gender	Age	Height	Weight	Side	Speed (m/s)	Foot-Off Event	Amplitude Range Stance Phase (in °)	Amplitude Range Swing Phase (in °)
$m_{1}$	F	20	1.61	65.0	L	1.23	63.1	[−1.8, 35.5]	[−5.4, 56.9]
$m_{2}$	F	21	1.68	60.0	L	1.11	63.2	[1.0, 35.7]	[−2.9, 63.5]
$m_{3}$	M	22	1.83	68.1	L	1.17	63.9	[6.1, 50.4]	[4.4, 68.4]

## Data Availability

Due to the nature of this research, participants of this study did not agree for their data to be shared publicly, so supporting data are not available.
